# Repetitive transcranial magnetic stimulation increases the brain’s drainage efficiency in a mouse model of Alzheimer’s disease

**DOI:** 10.1186/s40478-021-01198-3

**Published:** 2021-06-02

**Authors:** Yangyang Lin, Jian Jin, Rongke Lv, Yuan Luo, Weiping Dai, Wenchang Li, Yamei Tang, Yuling Wang, Xiaojing Ye, Wei-Jye Lin

**Affiliations:** 1 Department of Rehabilitation Medicine, the Sixth Affiliated Hospital, Sun Yat-sen University, Guangzhou, China; 2 Guangzhou Sport University, Guangzhou, China; 3 Guangdong Provincial Key Laboratory of Malignant Tumor Epigenetics and Gene Regulation, Guangdong-Hong Kong Joint Laboratory for RNA Medicine, Medical Research Center, Sun Yat-sen Memorial Hospital, Sun Yat-sen University, Guangzhou, China; 4 Guangdong Province Key Laboratory of Brain Function and Disease, Zhongshan School of Medicine, Sun Yat-sen University, Guangzhou, China; 5 Faculty of Forensic Medicine, Zhongshan School of Medicine, Sun Yat-sen University, Guangzhou, China; 6 Guangdong Province Translational Forensic Medicine Engineering Technology Research Center, Sun Yat-sen University, Guangzhou, China; 7 Department of Joint Surgery, the First Affiliated Hospital, Sun Yat-sen University, Guangzhou, China; 8 Department of Neurology, Sun Yat-sen Memorial Hospital, Sun Yat-sen University, Guangzhou, China

**Keywords:** Alzheimer’s disease, Repetitive transcranial magnetic stimulation, Meningeal lymphatics, Glymphatic system

## Abstract

Alzheimer’s disease (AD) is a progressive neurodegenerative disease with high prevalence rate among the elderly population. A large number of clinical studies have suggested repetitive transcranial magnetic stimulation (rTMS) as a promising non-invasive treatment for patients with mild to moderate AD. However, the underlying cellular and molecular mechanisms remain largely uninvestigated. In the current study, we examined the effect of high frequency rTMS treatment on the cognitive functions and pathological changes in the brains of 4- to 5-month old 5xFAD mice, an early pathological stage with pronounced amyloidopathy and cognitive deficit. Our results showed that rTMS treatment effectively prevented the decline of long-term memories of the 5xFAD mice for novel objects and locations. Importantly, rTMS treatment significantly increased the drainage efficiency of brain clearance pathways, including the glymphatic system in brain parenchyma and the meningeal lymphatics, in the 5xFAD mouse model. Significant reduction of Aβ deposits, suppression of microglia and astrocyte activation, and prevention of decline of neuronal activity as indicated by the elevated c-FOS expression, were observed in the prefrontal cortex and hippocampus of the rTMS-treated 5xFAD mice. Collectively, these findings provide a novel mechanistic insight of rTMS in regulating brain drainage system and β-amyloid clearance in the 5xFAD mouse model, and suggest the potential use of the clearance rate of contrast tracer in cerebrospinal fluid as a prognostic biomarker for the effectiveness of rTMS treatment in AD patients.

**Supplementary Information:**

The online version contains supplementary material available at 10.1186/s40478-021-01198-3.

## Introduction

Alzheimer’s disease (AD) is the most common type of dementia, featured by progressive impairment of cognitive functions across multiple domains including memory, language, emotion, executive ability, and eventually the ability to live independently [60]. Pathologically, AD is defined by the appearance of amyloid plaque and neurofibrillary tangles in the brain [42]. Despite traditionally being considered as a neurodegenerative disorder, emerging evidences suggest that dysregulations of non-neuronal cells in the brain may contribute to early pathological development of AD [6]. For example, overactivation of microglia and astroglia by amyloid-beta (Aβ) peptide-formed oligomers or amyloid plaques has been reported to result in glial activation, chronic inflammation and subsequent damages to neuronal synapses in the AD brains [1]. Aberrant angiogenesis has also been observed in AD patients and mouse models, likely to cause cerebral hypoperfusion and insufficient energy supply [3, 22]. Furthermore, recently discovered/rediscovered brain drainage machinery, the glymphatic system along the cerebral vasculatures in the brain parenchyma and the meningeal lymphatic vessels in the dura mater, have been proposed to play important roles in the clearance of Aβ from the brain, and aging-related impairment of the glymphatic system or the meningeal lymphatics is thought to aggravate Aβ accumulation in the AD brains [14, 29, 49], forming a vicarious feedback cycle to aggravate AD pathological development. Previous studies have shown the perivascular localization of astroglial water channel aquaporin-4 (AQP4) is critical for the influx and glymphatic transport of cerebrospinal fluid (CSF) [48], and is necessary for the clearance of interstitial Aβ and Tau by the glymphatic system [29, 30, 67]. Reduced perivascular astrocytic end feet localization of AQP4 and increased astrocyte activation in the aged or traumatic brain injury mouse models have been reported to exacerbate glymphatic pathway dysfunction [25, 28]. Given the complex nature of AD, advance in effective treatments is still lacking and will require the mechanistic elucidation of the disease and the multiple cell types that are involved.

Repetitive transcranial magnetic stimulation (rTMS) is a noninvasive therapy that uses rapidly changing magnetic field to modulate the electrical activity of the brain [63]. A large number of clinical studies have suggested rTMS as a promising treatment for mild and moderate AD [5, 55, 56, 63]. A wide array of rTMS protocols have been tested on early and middle stage AD patients. The rTMS is normally administered for 5–30 consecutive days, with treatment effects lasting for 4–12 weeks [9, 66]. It has been noticed that high frequency rTMS is more effective than low frequency rTMS for improving cognitive functions and recovering the daily living ability of AD patients [66, 69]. Furthermore, a meta-analysis on randomized-controlled clinical trials suggested that high frequency rTMS treatment that targeted multiple brain regions was more effective than targeting any single region for cognitive improvement in AD patients [41]. A few studies have shown that rTMS treatment reduces the production of Aβ peptide, recovers neuronal plasticity and reduces neuronal apoptosis in AD mouse models [7, 8, 27, 62]. Despite that, the cellular mechanisms by which rTMS improves cognitive functions and how rTMS may affect Aβ clearance in the AD brains remain under-investigated.

In the current study, we examined the effect of high frequency wide-field rTMS treatment on the cognitive functions and pathological changes of neurons and glia in the brains of 5xFAD mice, an Aβ precursor protein (APP)/presenilin-1 (PS1) double transgenic mouse model that develops rapid cerebral amyloid plaques and gliosis. We showed that rTMS treatment at early age of 5xFAD mice effectively prevented decline of long-term memories for novel object and location, which was accompanied by enhanced drainage efficiency through brain glymphatic system and meningeal lymphatics, reduction of Aβ deposits, reduced activation of microglia and astrocyte, and prevention of decline of neuronal activity as indicated by increased c-FOS expression in the 5xFAD mouse brains. Collectively, these findings provide novel mechanistic insights of rTMS for the treatment of early stage AD via improved clearance of Aβ deposits through brain glymphatic system and meningeal lymphatics. Our findings also suggest that improvement of CSF clearance efficiency, which can be measured by clinically available imaging techniques [4, 33, 39], may serve as a prognostic biomarker for the effectiveness of rTMS in AD patients.

## Materials and methods

### Animals

The 5xFAD mice (B6/SJL genetic background) overexpressing both human APP harboring the Swedish (K670N, M671L), Florida (I716V) and London (V717I) FAD mutations, and the PS1 harboring the two FAD mutations (M146L and L286V) were obtained from the Jackson Laboratory. 4–5 months old female and male 5xFAD mice and their wild-type littermates were used. The mice were housed in groups of 4–5 in an environmentally controlled animal facility on a 12 h light/dark cycle. Food and water were available ad libitum. All animal studies were approved by the Institutional Animal Care and Use Committee of the Sun Yat-sen University.

### rTMS procedures

For the delivery of rTMS, 5xFAD mice were placed in homemade cloth sleeves which gently and temporarily restrained their movement, with the top of the head exposed. The mice were breathing normally without visible struggling during rTMS stimulation. The rTMS was delivered by a magnetic stimulator (CCY-II, Wuhan Yiruide Medical Equipment, Wuhan, China) connected to a round coil (diameter: 6.5 cm). The head of mouse was pressed against the center of the coil. The rTMS was administered between 10 am to 12 pm for 14 consecutive days. On each day, the mice received 100 sessions of rTMS treatment with inter-session interval of 5 s. In each session, 40 burst trains of 20 Hz stimulation were delivered, with the magnetic stimulation intensity set at 1.38 T. The control wild-type and 5xFAD mice underwent the same procedures including restraint and being exposed to the noise from the magnetic stimulator, except that they were not placed under the coil.

### Novel object recognition (NOR) and novel object location (NOL) tasks

The mice were handled for five days before conducting behavioral training and test. Animal behavior was videotaped and scored by 2–3 independent investigators blind to the experimental conditions, or by the TopScan software (CleverSys, Reston, VA, USA). The NOR and NOL tests were modified based on previous studies [40, 64]. Briefly, the tests were carried out in a 40 × 40 × 40 cm arena placed in a quiet room with dim light. During the habituation session on the first day, the mice were allowed to explore the arena without objects for 10 min. On the second day, the mice explored the same arena again for 10 min, with two objects placed on the two ends of a side wall. On the third day, the mice were tested for their long-term memory for objects. During the test session, the mice were allowed to explore the arena for 5 min, with one of the old objects replaced by a new object. The NOL test was carried out following the same procedures of NOR, except that during the test session, one of the previously explored objects was placed to a new location across the arena. The discrimination index (DI) was calculated as follows: [(time exploring the novel object or the object placed to a novel location–time exploring the familiar) / (total time exploring both objects)] × 100.

### Open field test

We used the habituation session for NOR or NOL test as the open field test for accessing the exploratory and anxiety-like behaviors of the mice, as previous described [37]. The exploratory activity was measured by the total distance of mice exploring the arena. The anxiety-like behaviors were accessed by the distance and time spent in the 20 × 20 cm center zone as percentages of the total distance and time, respectively.

### Y-maze task

Working memory was measured by spontaneous alternation in a Y-maze as previously described [38]. The Y-maze consisted of 3 arms of 50 × 10 × 20 cm each. The mice were placed in the center zone of the maze and allowed to freely explore the maze for 5 min. An alternation event was defined as the completion of sequential entries into all three arms. Percent alternation was calculated as follows: [(number of alternations)/(total entries -2)] × 100.

### Intracisternal injection

Intracisternal injection was carried out as described previously [14]. Briefly, the mice were anesthetized by intraperitoneal injection of 5% chloral hydrate in saline (0.1 mL per 10 g body weight). The hair on the neck was shaved and the head was fixed on a stereotaxic apparatus. The skin on the neck was then incised, and the muscle layers were retracted to expose the cisterna magna. Using an infusion pump (Model R452, RWD Life Science, China) with a Hamilton syringe connected to a 30-gauge needle, 5μL of 10kD Dextran-Alexa Fluor 647 (Thermo Fisher, 1% in artificial cerebrospinal fluid) was injected at a rate of 1μL per min. The needle was left in place for an additional 10 min after injection to prevent backflow. The neck skin was then sutured. The mice were placed on a heating pad to maintain body temperature and sacrificed 20 min after withdrawal of the injection needles.

### Immunofluorescence staining and image analysis

At the aforementioned time point after tracer injection, mice were transcardially perfused with PBS followed by 4% paraformaldehyde in PBS. The brains were harvested and post-fixed in PBS with 4% paraformaldehyde at 4 °C overnight. 40 μm coronal sections were collected by a cryostat for free-floating immunofluorescence staining. Two sections of the prefrontal cortex (mPFC) brain region at approximately 1.78 mm and 1.98 mm anterior to the Bregma, and two sections of the dorsal hippocampus (dHC) brain region and the primary sensory cortex (S1) at approximately 1.58 mm and 1.82 mm posterior to the Bregma were sampled for each staining. The averaged values from the two sections of the same animals were taken as one data point for each staining. The sections were incubated in the blocking buffer (5% normal goat serum, 1% bovine serum albumin in PBS with 0.4% Triton X-100) for 2 h at room temperature, and stained with primary antibodies diluted in the blocking buffer for approximately 40 h at 4 °C. The primary antibodies used in this study include: anti-GFAP (Cell Signaling Technologies, cat#: 3670S, 1:500), anti-Aβ42 (BioLegend, cat#: 803001, 1:2000), anti-NeuN (Cell Signaling Technology, cat#: 24307S, 1:500), anti-IBA1 (Fujifilm, cat#: 019-19741, 1:500), anti-c-FOS (Cell Signaling, cat#: 2250, 1:750), anti-AQP4 (Millipore, cat#: AB3594, 1:400), and anti-LYVE1 (Abcam, cat#: ab14917, 1:400). After 3 × 10 min washes in PBS with 0.4% TritonX-100, the sections were stained with secondary antibodies diluted in the blocking buffer for 2 h at room temperature, followed by additional washes before mounting onto glass-slides. For whole mount meninges staining, after perfusion, the meninges were postfixed in PBS with 2% paraformaldehyde at 4 °C overnight, and stained with primary antibodies overnight at 4 °C. Deep cervical lymph nodes were also harvested after perfusion, post-fixed in PBS with 4% paraformaldehyde at 4 °C overnight, and afterwards 30 μm sections were collected and mounted on glass-slides. The boarder of indicated brain areas was defined based on the mouse atlas of the 2nd edition of “the Mouse Brain in Stereotaxic Coordinates” published by Paxinos and Franklin [53]. The schematic diagram of dura mater was referenced based on previous report [54]. Images were taken by an epi-fluorescent microscope (Nikon Eclipse Ni-U) or a confocal microscope (Zeiss LSM800), and analyzed by ImageJ (version 1.52p, NIH, US). For the analysis of intraneuronal Aβ, we did immunofluorescence co-staining of neuronal marker NeuN with Aβ antibody (6E10) and only analyzed Aβ fluorescence signals within the NeuN-positive area. For the calculation of cell density, we measured the total cell number in specific brain regions and divided the number by the area size. For tracer analysis, the fluorescence intensity (arbitrary units) of intracisternally injected tracer (10 kDa Dextran-Alexa Fluor 647) within the mPFC, SSS + TS areas of dura mater, or dCLN was measured, and total intensity of tracer was divided by the area of region of interest [29, 44]. For quantification of aquaporin 4 (AQP4) polarity in the medial prefrontal cortex, we used high-stringency threshold of ImageJ to define the perivascular end feet area of AQP4 staining (high AQP4 staining intensity) as described previously [25]. AQP4 polarity was calculated as follows: [(fluorescence intensity of perivascular end AQP4)/(fluorescence intensity of total AQP4)].

### Statistical analyses

Data were presented as mean ± SEM. Statistical analyses were carried out using Graphpad Prism (version 8, GraphPad Software, San Diego, CA, USA). One-way ANOVA followed by Tukey's multiple comparisons test were used to compare multiple groups. Unpaired two-tailed Student’s *t*-test was used to compare two groups. A *p* value of less than 0.05 was considered statistically significant.

## Results

### rTMS prevents the loss of long-term memory in 5xFAD mice

**Fig. 1:**
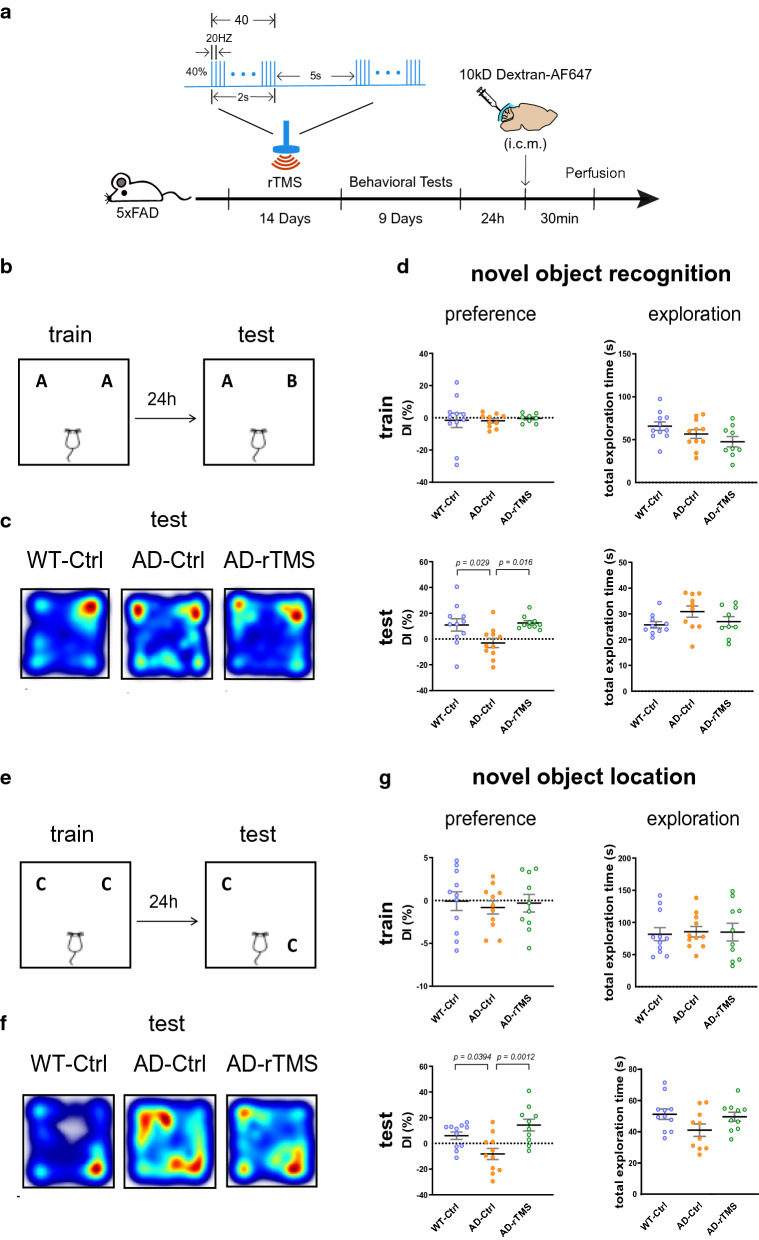
rTMS prevents the loss of long-term memories of 5xFAD mice for novel objects and locations. **a** The experimental timeline of rTMS treatment, behavioral tests, injection of 10kD Dextran tracer into the cisterna magna (i.c.m.), and perfusion. **b** Schematic of the novel object recognition task. A and B indicates different objects. **c** Representative heatmaps of animals’ paths during the test session of the novel object recognition task. **d **The discrimination index (DI%) and the total time spent on exploring both objects (total exploration time) during the training and the test sessions in the novel object recognition task (n = 10–11 mice per group). **e** Schematic of the novel object location task. Both Cs are identical objects. **f** Representative heatmaps of animals’ paths during the test session of the novel object location task. **g** The discrimination index (DI%) and the total time spent on exploring both objects (total exploration time) during the training and the test sessions in the novel object location task (n = 10–11 mice per group). All data are presented as mean ± SEM and analyzed by one-way ANOVA followed by Tukey's multiple comparisons test. WT-Ctrl: wildtype littermates received sham treatment, AD-Ctrl: 5xFAD mice received sham treatment, AD-rTMS: 5xFAD mice received rTMS treatment

We first evaluated the therapeutic effects of rTMS on the cognitive functions of 5xFAD mice, a familial AD mouse model which overexpresses five familial AD mutations on human APP and PSEN1 genes. The 5xFAD mice develop aggressive amyloid pathology and showed cognitive deficits across multiple domains around 4–5 months of age [18, 24, 36, 52]. To examine if rTMS treatment improved memory performance of 5xFAD mice, high frequency rTMS (20 Hz) with magnetic stimulation intensity at 1.38 T was delivered to mice for 14 consecutive days, followed by behavioral tests including novel object recognition and novel object location tasks (Fig. 1a). The parameters and stimulus intensity of rTMS protocol used in this study was similar to that used in a clinical study by Cotelli et al., which showed beneficial effects of rTMS on language performance in AD patients [10]. We found that rTMS treatment significantly prevented the decline of long-term memories for both object identity and locations in 5xFAD mice at 4–5 months of age (Fig. 1b–g).

Working memory deficit has been reported previously in 5xFAD mice [17, 18, 52]. We then used Y-maze to assess the effects of rTMS on the spatial working memory of 5xFAD mice. Our data showed a trend towards impaired spatial memory of 5xFAD mice, which however was not improved by rTMS treatment (Additional file 1: Supplementary Fig. 1a). Moreover, increased anxiety has been reported in AD patients, though the reports from AD mouse models are still controversial, possibly due to a variety of factors including the dosage of transgenes and genetic backgrounds of the mice [20, 31, 34, 51]. In our study, we observed that 5xFAD mice with sham treatment showed increased anxiety-like behaviors, as demonstrated by avoiding traveling to the center zone in an open field, which was not improved, however, by rTMS treatment (Additional file 1: Supplementary Fig. 1b-c). Notably, rTMS treatment significantly decreased the locomotion of 5xFAD mice in the open field test (Additional file 1: Supplementary Fig. 1c).

Taken together, our findings confirmed that the 20 Hz rTMS treatment prevented decline of long-term memory performance but was not effective in improving impaired spatial working memory or increased anxiety of 5xFAD mice.

### rTMS decreases Aβ accumulation in the 5xFAD brains

**Fig. 2:**
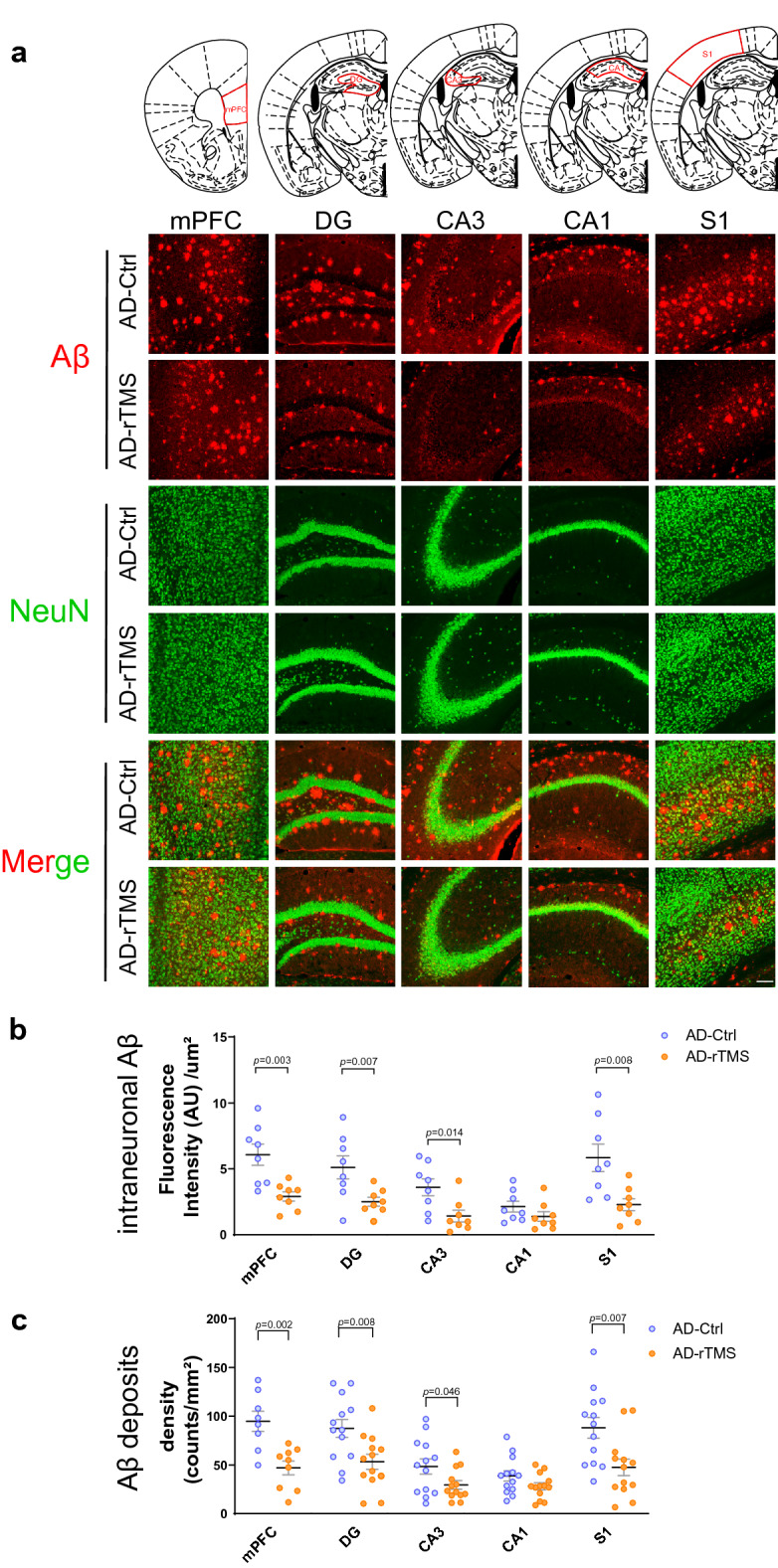
rTMS decreases Aβ accumulation in the 5xFAD brains. **a** Representative images of Aβ and NeuN immunofluorescence staining in the mPFC, the DG, CA3 and CA1 subregions of dorsal hippocampus and the S1 cortex. Coronal positions of the brain sections shown on the top corresponds to the immunofluorescence staining results below. Red: 6E10 staining. Green: NeuN staining. Scale bar: 200 μm. **b** Quantitative analyses of Aβ fluorescence intensity within the NeuN-positive cells (intraneuronal Aβ) across different regions, comparing 5xFAD mice received sham treatment (AD-Ctrl) and 5xFAD mice received rTMS treatment (AD-rTMS) (n = 8–13 mice per group). **c** Quantitative analyses of the density of extracellular plaque-like Aβ deposits across different regions, comparing AD-Ctrl and AD-rTMS groups (n = 8–13 mice per group). All data are presented as mean ± SEM and analyzed by unpaired two-tailed Student’s *t*-test. mPFC: medial prefrontal cortex, DG: dentate gyrus, CA3: cornu ammonis 3, CA1: cornu ammonis 1, S1: primary sensory cortex

Accumulation of amyloid plaque in the brain is a pathological hallmark for AD [42, 60]. The 5xFAD mice rapidly develop amyloid pathology at the age of 2 months [52]. Since rTMS effectively prevented the loss of long-term memory in 5xFAD mice, we wondered whether such change was accompanied by a decrease of parenchymal Aβ deposits. We examined the medial prefrontal cortex (mPFC) and the dorsal hippocampus (dHC), two regions whose dysfunction are critically involved in the cognitive impairment during AD progression [23, 24, 59]. As Aβ deposits have been reported to appear early in the deep layers of the primary sensory cortex (S1) of 5xFAD mice [52], we also included the S1 cortex in our analyses. Consistent with previous reports [52], accumulation of plaque-like Aβ deposits were detected in all regions, with most robust deposits observed in the mPFC, the dentate gyrus (DG) of dHC and the S1 cortex (Fig. 2a). rTMS treatment significantly reduced both intraneuronal Aβ and plaque-like Aβ deposits in the mPFC, DG and cornu ammonis 3 (CA3) of the dHC and the S1 cortex as compared with untreated 5xFAD mice (Fig. 2b–c). There was also a trend towards reduction of intraneuronal Aβ and Aβ deposits in the cornu ammonis 1 (CA1) of the dHC after rTMS treatment (Fig. 2b–c). These data suggest that rTMS treatment effectively alleviated the development of pathological Aβ deposits in the 5xFAD brains.

### rTMS improves the drainage efficiency of the glymphatic/meningeal lymphatic systems

**Fig. 3:**
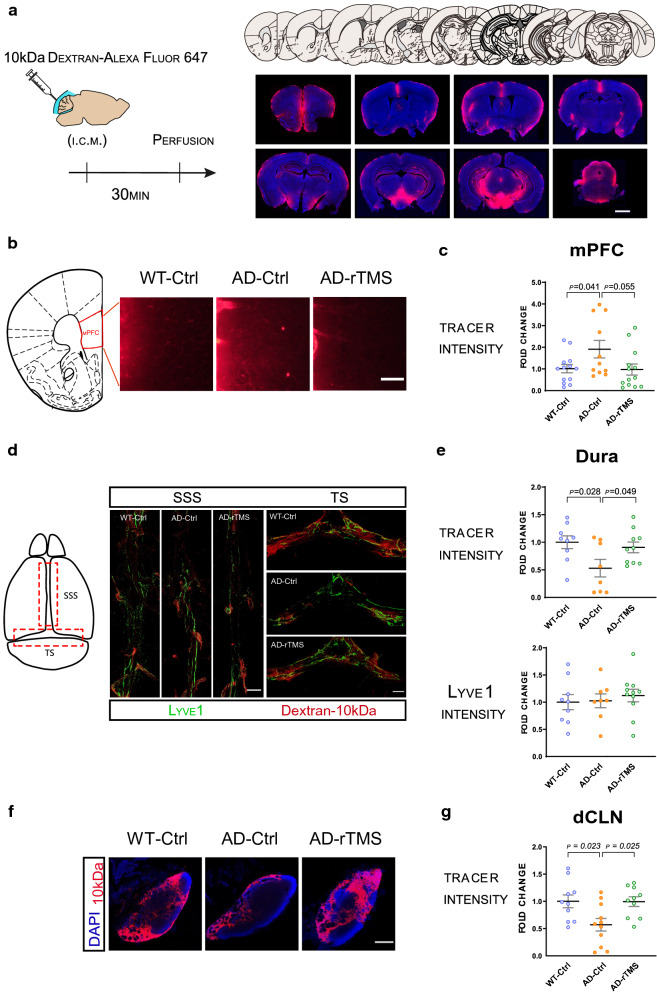
Reduced deterioration in the drainage efficiency of glymphatic system and meningeal lymphatics in the 5xFAD mice treated with rTMS. **a** Left: Schematic of the injection of 10 kDa Dextran-Alexa Fluor 647 tracer into cisterna magna (i.c.m.) and timeline of perfusion. Right: Representative images of tracer (red) distribution and DAPI staining (blue) in the wildtype brain parenchyma. Coronal positions of the brain sections shown on the top corresponds to the tracer staining results below. Scale bar: 100 μm. **b** The atlas of the brain coronal section containing mPFC, and the representative images of the tracer (red) in the mPFC of different groups. Scale bar: 200 μm. **c** Quantitative analyses of the fluorescence intensity of the tracer in the mPFC across groups. (n = 8–12 mice per group). **d** Left: the schematic diagram of dura mater, with dotted red line bordering the superior sagittal sinus (SSS) area as well as the transverse sinus (TS) area chosen for image analyses. Right: the representative images of Lyve1 staining (green) and the tracer (red) in the SSS and TS of the dura mater. Scale bar: 450 μm. **e** Quantitative analyses of the fluorescence intensity of the tracer and Lyve1 staining in the dura mater (SSS + TS areas) across groups (n = 7–10 mice per group). **f** Representative images of the tracer (red) and DAPI (blue) in the deep cervical lymph nodes (dCLN). Scale bar: 150 μm. **g** Quantitative analyses of the fluorescence intensity of the tracer in the dCLN across groups (n = 10–11 mice per group). All data are presented as mean ± SEM of the fold change of the WT-Ctrl group and analyzed by one-way ANOVA followed by Tukey's multiple comparisons test. WT-Ctrl: wildtype littermates received sham treatment, AD-Ctrl: 5xFAD mice received sham treatment, AD-rTMS: 5xFAD mice received rTMS treatment

The reduction of Aβ deposits by rTMS could be mediated by two processes: reduced Aβ production and/or facilitated Aβ clearance. Indeed, consistent with previous reports [8, 47, 65], we observed a significant reduction in the intraneuronal Aβ levels in the mPFC, dHC and S1 cortex, indicating that rTMS treatment suppressed Aβ production (Fig. 2c). Could rTMS also facilitate Aβ clearance? The recently discovered/rediscovered glymphatic system in the brain parenchyma, and the meningeal lymphatics in the dura mater, which further connect to the deep cervical lymph nodes (dCLNs), are important paths for removing macromolecules from the brain [13, 45]. To examine and evaluate the drainage efficiency of the glymphatic system and meningeal lymphatics in the 5xFAD mouse brains as demonstrated previously [14, 29], we injected 10 kD Dextran tracer conjugated with Alexa Fluor 647 into the cisterna magna. Mice were sacrificed 30 min later and the brain parenchyma, the meninges, and the dCLNs were collected for analysis. In the wild-type mice, the fluorescent tracer was found accumulated in the mPFC, hypothalamus and mid-brain regions, with relatively low amount of tracer observed in the dHC and the S1 cortex (Fig. 3a). We therefore focused on the mPFC for the subsequent analyses of tracer distribution in rTMS- and sham-treated 5xFAD mice. Compared with the wild-type littermates, the amount of tracer trapped in the mPFC of 5xFAD mice was significantly elevated, whereas rTMS treatment ameliorated the accumulation of tracer (Fig. 3b–c). As the glymphatic system connects to the meningeal lymphatics, to examine whether the accumulated tracer in the mPFC of 5xFAD brains may be due to reduced drainage efficiency of the glymphatic system and meningeal lymphatics, we examined the distribution of tracer in the dura mater where meningeal lymphatics were identified, and in the dCLNs which connect to meningeal lymphatics [2, 46]. Significant reduction of tracer was detected in the dura mater and the dCLNs of the 5xFAD mice as compared to their wild-type littermates, whereas rTMS treatment significantly prevented such changes (Fig. 3d–g). Notably, reduced drainage efficiency in the 5xFAD was not associated with the expression level or polarization of Aquaporin-4 (AQP4), a known regulator of the glymphatic system, in the mPFC (Additional file 2: Supplementary Fig. 2). There was also no significant difference in the expression level of meningeal lymphatic marker Lyve1 among wild type, rTMS-treated and non-treated 5xFAD mice, suggesting that rTMS enhanced the function of glymphatic system and meningeal lymphatics without inducing lymphagenesis (Fig. 3d, e).

Collectively, our findings indicate that rTMS treatment may reduce Aβ deposits by improving the drainage efficiency of Aβ by the glymphatic system and meningeal lymphatics in the 5xFAD brains.

### rTMS reduces glial activation and prevents the decline of neuronal activity in the 5xFAD brains

**Fig. 4:**
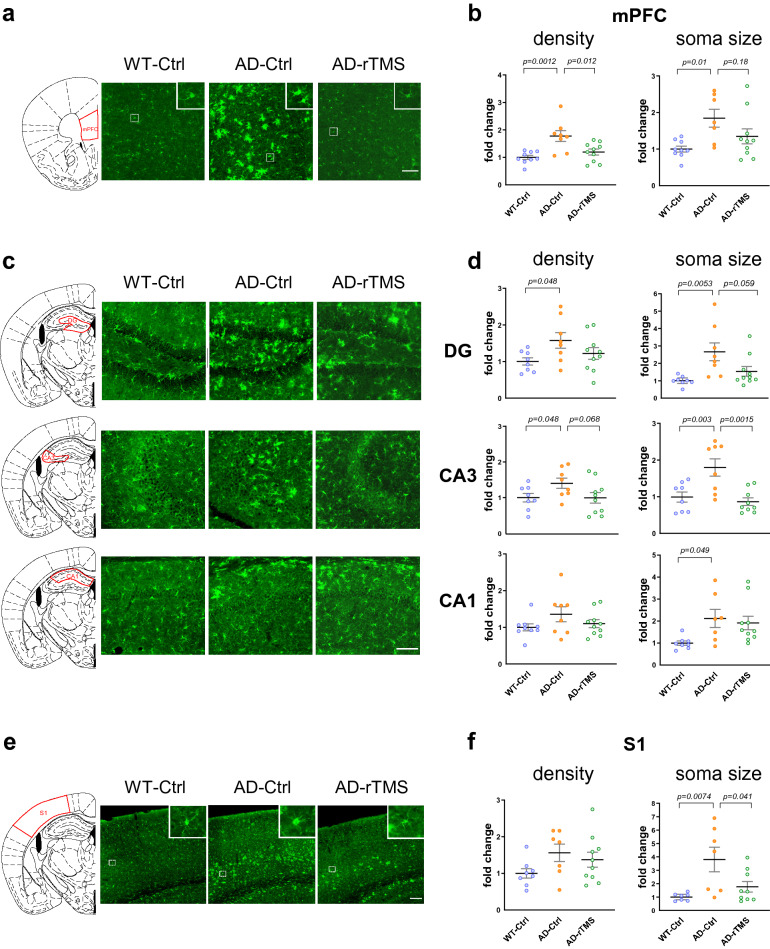
rTMS decreases microglial density and soma size in the 5xFAD brains. **a** Representative images of the microglia marker IBA1 staining in the mPFC. Top right insert: enlarged images demonstrating the soma size of the microglia. Scale bar: 100 μm. **b** Quantitative analyses of the density and the soma size of IBA1-labelled microglia in the mPFC across groups (n = 8–10 mice per group, 124 ± 4 cells per section were sampled for the analysis). **c.** Representative images of the microglia marker IBA1 staining in different subregions of dorsal hippocampus. Top right insert: enlarged images demonstrating the soma size of the microglia. Scale bar: 100 μm. **d** Quantitative analyses of the density and the soma size of IBA1-labelled microglia in different subregions of hippocampus across groups (n = 8–10 mice per group, 154 ± 7, 134 ± 16 and 200 ± 28 cells were sampled from DG, CA3 and CA1 brain regions for analysis). **e** Representative images of the microglia marker IBA1 staining in the S1 cortex. Top right insert: enlarged images demonstrating the soma size of the microglia. Scale bar: 100 μm. **f** Quantitative analyses of the density and the soma size of IBA1-labelled microglia in the S1 cortex across groups (n = 7–10 mice per group, 554 ± 60 cells per section were sampled for analysis). All data are presented as mean ± SEM of the fold change of the WT-Ctrl group and analyzed by one-way ANOVA followed by Tukey's multiple comparisons test. WT-Ctrl: wildtype littermates received sham treatment, AD-Ctrl: 5xFAD mice received sham treatment, AD-rTMS: 5xFAD mice received rTMS treatment. mPFC: medial prefrontal cortex, DG: dentate gyrus, CA3: cornu ammonis 3, CA1: cornu ammonis 1, S1: primary sensory cortex

**Fig. 5:**
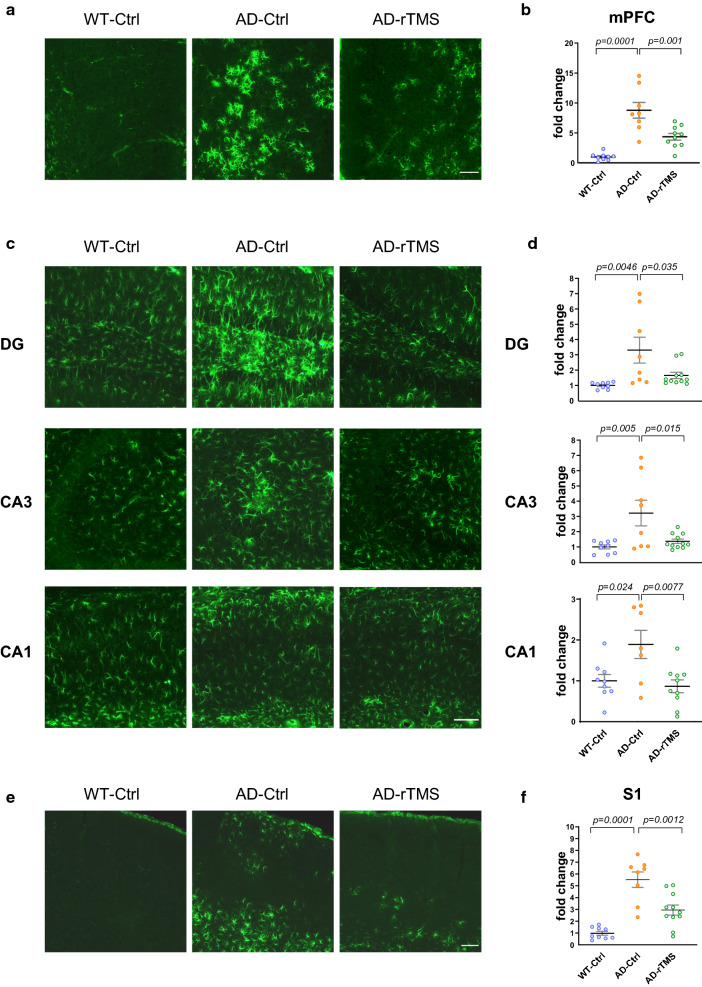
rTMS reduces activation of astrocytes in the 5xFAD brains. **a** Representative images of GFAP staining in the mPFC. Scale bar: 100 μm. **b** Quantitative analyses of the fluorescence intensity (normalized to the area of region-of-interest) of GFAP staining in the mPFC across groups (n = 8–10 mice per group). **c** Representative images of GFAP staining in different subregions of the dorsal hippocampus. Scale bar: 100 μm. **d** Quantitative analyses of the fluorescence intensity (normalized to the area of region-of-interest) of GFAP staining in different subregions of the hippocampus across groups (n = 8–11 mice per group). **e** Representative images of GFAP staining in the S1 cortex. Scale bar: 150 μm. **f** Quantitative analyses of the fluorescence intensity (normalized to the area of region-of-interest) of GFAP staining in the S1 cortex across groups (n = 8–11 mice per group). All data are presented as mean ± SEM of the fold change of the WT-Ctrl group and analyzed by one-way ANOVA followed by Tukey's multiple comparisons test. WT-Ctrl: wildtype littermates received sham treatment, AD-Ctrl: 5xFAD mice received sham treatment, AD-rTMS: 5xFAD mice received rTMS treatment. mPFC: medial prefrontal cortex, DG: dentate gyrus, CA3: cornu ammonis 3, CA1: cornu ammonis 1, S1: primary sensory cortex

Aβ deposits have been shown to induce glial activation as indicated by increased microglial number and soma size, as well as increased Glial Fibrillary Acidic Protein (GFAP) expression in astrocytes in both AD patients and mouse models [26]. As we found rTMS effectively reduced Aβ deposits in multiple brain regions of 5xFAD mice, we further examined whether such changes were accompanied with altered activation of microglia and astrocytes. Consistent with previous reports [52], activation of microglia indicated by increase in cell number and soma size of ionized calcium-binding adapter molecule 1 (IBA1)-positive cells was observed in the mPFC, hippocampal CA3 and S1 cortex of 5xFAD mice at 4–5 months of age, which was reduced by rTMS intervention (Fig. 4, density of IBA1-positive cells, mPFC, WT-Ctrl: 203 ± 15 cells/mm^2^; DG, WT-Ctrl: 286 ± 21 cells/mm^2^; CA3, WT-Ctrl: 254 ± 24 cells/mm^2^; CA1, WT-Ctrl: 231 ± 19 cells/mm^2^; S1 cortex, WT-Ctrl: 273 ± 29 cells/mm^2^; soma size of IBA1-positive cells, mPFC, WT-Ctrl: 48 ± 3 μm^2^; DG, WT-Ctrl: 65 ± 6 μm^2^; CA3, WT-Ctrl: 61 ± 6 μm^2^; CA1, WT-Ctrl: 55 ± 3 μm^2^; S1 cortex, WT-Ctrl: 62 ± 6 μm^2^). Similarly, activation of GFAP-positive astrocytes was shown in the mPFC, dHC and S1 cortex of 5xFAD mice, which was also reduced by rTMS (Fig. 5). These data therefore suggest that rTMS treatment alleviated microglia and astrocyte activation in the brain parenchyma of 5xFAD mice.

**Fig. 6:**
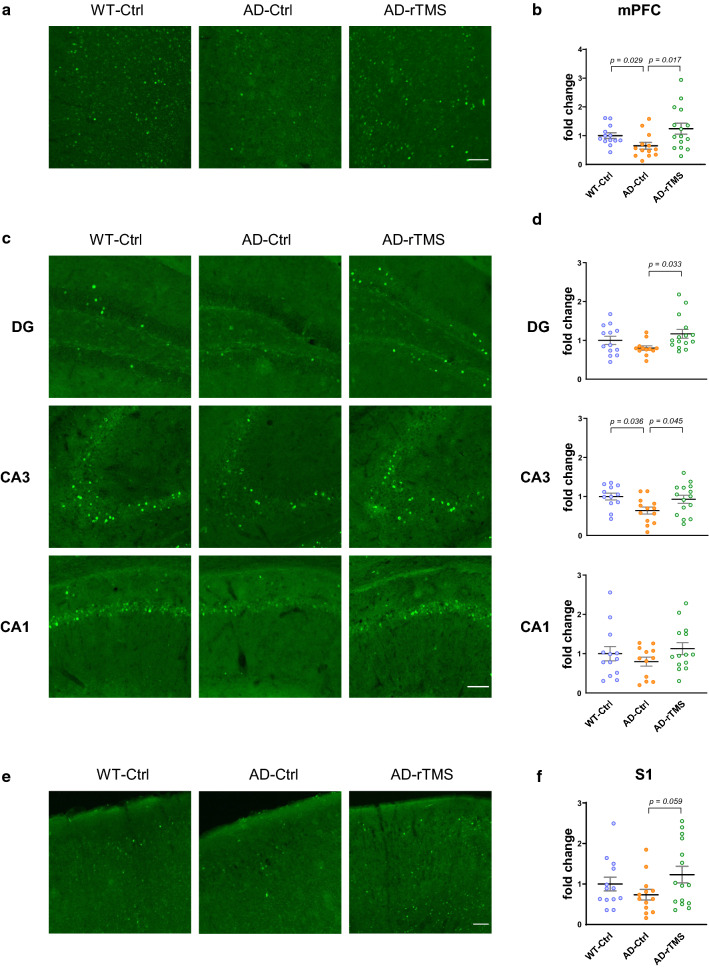
rTMS prevents decline of neuronal activity in the 5xFAD brains. **a** Representative images of c-FOS staining in the mPFC. Scale bar, 100 μm. **b** Quantitative analyses of the density of c-FOS positive cells in the mPFC across groups (n = 8–10 mice per group, 98 ± 16 cells per section were sampled for the analysis). **c** Representative images of c-FOS staining in different subregions of dorsal hippocampus. Scale bar, 100 μm. **d** Quantitative analyses of the density of c-FOS positive cells in different subregions of hippocampus across groups (n = 8–11 mice per group, 95 ± 6, 89 ± 22 and 95 ± 21 cells were sampled from DG, CA3 and CA1 brain regions for analysis). **e** Representative images of c-FOS staining in the S1 cortex. Scale bar, 150 μm. **f** Quantitative analyses of the density of c-FOS positive cells in the S1 cortex across groups (n = 8–11 mice per group, 414 ± 28 cells per section were sampled for the analysis). All data are presented as mean ± SEM of the fold change of the WT-Ctrl group and analyzed by one-way ANOVA followed by Tukey's multiple comparisons test. WT-Ctrl: wildtype littermates received sham treatment, AD-Ctrl: 5xFAD mice received sham treatment, AD-rTMS: 5xFAD mice received rTMS treatment. mPFC: medial prefrontal cortex, DG: dentate gyrus, CA3: cornu ammonis 3, CA1: cornu ammonis 1, S1: primary sensory cortex

Lastly, since neuronal hypoactivity has been reported in memory-associated brain regions in the AD mouse models [11, 36], we wondered whether the aforementioned changes by rTMS treatment may prevent the decline of neuronal activity in the 5xFAD brains. As compared to the wild-type littermates, we found that the number of c-FOS-positive neurons (as an indicator of neuronal activity [21, 61]) was significantly decreased in the mPFC and the CA3 sub-region of dHC, and a trend towards decreased c-FOS expression was also observed in the DG and S1 cortex of 5xFAD mice (Fig. 6, density of c-FOS-positive cells, mPFC, WT-Ctrl: 178 ± 16 cells/mm^2^; DG, WT-Ctrl: 98 ± 8 cells/mm^2^; CA3, WT-Ctrl: 102 ± 7 cells/mm^2^; CA1, WT-Ctrl: 91 ± 11 cells/mm^2^; S1 cortex, WT-Ctrl: 154 ± 23 cells/mm^2^). Notably, rTMS treatment significantly increased c-FOS-positive neurons in the affected brain regions (Fig. 6). Therefore, our findings revealed the decrease in neuronal activity was effectively prevented by rTMS intervention in the 5xFAD brains.

## Discussion

In the current study, we provided new evidences showing that early intervention by rTMS treatment could effectively reduce the loss of long-term memory performance and alleviate AD-related pathological development, including Aβ deposition and glial activation, in the 5xFAD mouse model. Importantly, our findings indicate that such improvement may be mediated by the increased drainage efficiency of brain parenchyma through the glymphatic system and meningeal lymphatics. Based on our findings, we proposed a new mechanistic model that rTMS may reduce the development of Aβ deposits by facilitating Aβ clearance along with reduced Aβ production, and together with the alleviation of glial activation, result in the prevention of further decline of neuronal activity and cognitive function.

The recently discovered/rediscovered glymphatic system in brain parenchyma and the meningeal lymphatics are considered as major pathways for clearance of toxic molecules from the brain. The glymphatic system was reported by Iliff et al. [29] as an interstitial compartment surrounding the cerebral vasculature and aligned by astrocyte end-feet. The polarized distribution of AQP4 protein in astrocytic end-feet is critically implicated in regulating glymphatic function, and Aqp4 knockout mice showed impairment in efflux of intracerebral injected Aβ, suggesting an important role of the glymphatic system in mediating Aβ clearance [29]. The glymphatic system further connects to the meningeal lymphatics in the dura mater, which tunnels down to peripheral dCLNs [2, 45, 46]. It has been previously proposed that Aβ may be transported to the subarachnoid space through the intracerebral glymphatic system and then through the meningeal lymphatics to the deep cervical lymph nodes [14, 29, 43]. In agreement with this model, disruption of meningeal lymphatics was reported to enhance Aβ accumulation in the brain parenchyma and dura mater of 5xFAD mice, supporting the idea that the brain drainage system plays an important role in parenchymal Aβ clearance and its impairment aggravates the development of Aβ-related pathology [15].

Our findings are in agreement with previous reports, that rTMS treatment resulted in the reduction of Aβ deposit in the hippocampus of AD mouse models [8, 47, 65]. Although previous studies have shown that rTMS may suppress the expression of APP and APP cleavage enzyme, β-secretase 1 (BACE1), therefore reduce the production and processing of Aβ in the AD mouse brains [27], however, merely reducing Aβ production may not be sufficient for the pathological improvement and cognitive benefits observed in rTMS-treated AD animal models and patients. Our results now provide evidences showing that, concomitant with reduced Aβ deposits in multiple brain regions as compared with untreated 5xFAD mice, two weeks of high frequency rTMS regime also significantly prevented the decline of cognitive function, likely through the improved drainage efficiency through the glymphatic system and meningeal lymphatics, which may facilitate the clearance of interstitial Aβ as suggested by recent studies [14, 16, 29, 30, 67]. Therefore, the therapeutic effects of rTMS on preventing the progression of Aβ pathology in the AD brains are likely two-fold: on one hand by suppressing Aβ production, and on the other hand by enhancing clearance of extracellular Aβ, rendering it an effective treatment for early stage AD. Further examinations of the promoting effects of rTMS on Aβ clearance may be tested directly by comparing the Aβ levels in deep cervical lymph nodes of AD mice with or without rTMS treatment, or indirectly by measuring the clearance rate of tracer-labeled Aβ peptides injected into AD mouse brains with or without rTMS treatment by in vivo two photon imaging [25]. It remains to be determined the molecular mechanisms of how drainage efficiency is regulated by rTMS in the AD brains.

Our study showed that in the mPFC of 5xFAD mice, significantly increased accumulation of 10 kDa Dextran-Alexa Fluor 647 was observed at 30 min after a single intracisternal injection of the fluorescent tracer. While such differences may be interpreted as a result of either impaired efflux or enhanced influx of the fluorescent tracer, however, our data demonstrated the significantly increased tracer in the brain parenchyma of 5xFAD mice was concomitant with reduced tracer in the meningeal lymphatics and dCLNs, suggesting impaired efflux of the drainage system in the 5xFAD brains. Of note, previous reports on the distribution of intracisternally injected tracer in the AD mouse models have been mixed [15, 29]. The discrepancy may be due to the distinct time points examined after tracer injection, and different types of tracers used in other studies. Moreover, often only brain parenchyma or meninges/dCLNs were investigated for the distribution of intracisternally injected tracer, thus lacking a comprehensive information about the kinetics of drainage efficiency between the AD and normal brains. To further evaluate functional changes of parenchymal drainage system in the AD, whole brain time-lapsed imaging may help to resolve the issues and discrepancies.

Neuroinflammation has been considered the main contributor to progressive neural damage and blood–brain barrier disruption in neurodegenerative diseases [1]. Our findings demonstrated the regulatory effects of rTMS on suppressing glial activation in the 5xFAD mouse brains, which may be explained by direct modulatory effect of rTMS on glial activities, or by the indirect consequences of rTMS on the reduction of Aβ plaques or the release of neurotransmitters from activated neurons[12, 50]. Previous findings have shown that rTMS may suppress or induce glial activation via different frequency and strength used and may be context-dependent. For example, rTMS treatment (gamma oscillations, 30-40 Hz) for 4 weeks has been reported to attenuate cuprizone-induced microglia activation and pro-inflammatory cytokine expression in the frontal cortex and hippocampus of the affected mouse brains [68]. In a rat model of spinal cord injury, high frequency (25 Hz) rTMS treatment for 8 weeks was found to suppress astrocyte activation [35]. On the contrary, a transient increase of astroglial GFAP expression has been reported in the ischemic injury rat model by 50 Hz rTMS treatment for 7 days, and in the demyelination lesion Mongolian gerbil model by 1 Hz rTMS for 14 days [19, 57]. The different effects of rTMS on glial activation and neuroinflammation therefore suggest the indirect regulatory mechanisms through other rTMS-influenced cells in the affected area. Whether it is the reduction of Aβ plaques or some other factors driven by the rTMS treatment that lowered glial activation and improved neuronal activity and cognition remains to be determined.

## Conclusions

In summary, our data suggested that rTMS alleviated the pathological changes and cognitive impairment in 5xFAD mice, likely through enhanced drainage efficiency of the glymphatic system and meningeal lymphatics, which in turn facilitated toxic Aβ removal and suppression of glial activation. The effectiveness of high-frequency rTMS treatment has been examined in clinical studies. Notably, patients diagnosed with early stage AD received rTMS stimulation bilaterally to the dorsal lateral prefrontal cortex showed cognitive improvement relative to their baseline state while patients with sham treatment showed no improvement [32, 58]. Together with our findings that rTMS treatment prevented the cognitive decline in 5xFAD mice, we hypothesize that the still limited accumulation of toxic Aβ, neuroinflammation and neuronal damage at early stage of AD may allow for better therapeutic effect of rTMS. It is also worth noting that the clearance efficiency of the glymphatic/meningeal lymphatic systems can now be measured by clinically available imaging techniques like intrathecal injection of Gadobutrol-based contrast tracer in combination with dynamic contrast enhanced magnetic resonance imaging techniques [4, 33, 39], therefore could serve as the prognostic marker of the disease and the effectiveness of rTMS treatment. Further exploration of the mechanistic targets of rTMS is warranted for its therapeutic potential for patients with AD and other neuropsychiatric disorders.

## Supplementary Information

**Additional file 1: Supplementary Fig. 1.** The effect of rTMS on spatial working memory and anxiety-like behaviors. **a**. Left: schematic of the Y-maze task. A, B and C are the 3 arms of the Y-maze. Right: Quantitative analyses of the spontaneous alternation (% alternation) and the number of total arm entries across groups (n = 10-11 mice per group). **b**. Representative heatmaps of animals’ paths in the open field test. **c**. Quantitative analyses of the time spent in the center zone as percentage of the total time, the distance travelled in the center zone as percentage of the distance travelled, and the total distance travelled in the open field across groups (n = 11-12 per group). All data are presented as mean ± SEM and analyzed by one-way ANOVA followed by Tukey's multiple comparisons test. WT-Ctrl: wildtype littermates received sham treatment, AD-Ctrl: 5xFAD mice received sham treatment, AD-rTMS: 5xFAD mice received rTMS treatment.

**Additional file 2: Supplementary Fig. 2.** rTMS does not change AQP4 expression or polarization in the mPFC. **a**. Representative images of AQP4 staining in the medial prefrontal cortex (mPFC). Scale bar: 150 μm. **b**. Quantitative analyses of the fluorescence intensity (normalized to the area of mPFC) of AQP4 staining and AQP4 polarity (defined as the fluorescence intensity of AQP4 staining on the perivascular end divided by the fluorescence intensity of total AQP4 staining) in the mPFC across groups (n = 8-9 mice per group). All data are presented as mean ± SEM of the fold change of the WT-Ctrl group and analyzed by one-way ANOVA followed by Tukey's multiple comparisons test. WT-Ctrl: wildtype littermates received sham treatment, AD-Ctrl: 5xFAD mice received sham treatment, AD-rTMS: 5xFAD mice received rTMS treatment.

## Data Availability

The datasets used and/or analyzed during the current study are available from the corresponding author on reasonable request.

## Abbreviations

AD: Alzheimer’s disease
Aβ: Amyloid beta
APP: Amyloid-beta precursor protein
PS1: Presenilin-1
BACE-1: β-Secretase 1
rTMS: Repetitive transcranial magnetic stimulation
NOR: Novel object recognition
NOL: Novel object location
DI: Discrimination index
mPFC: Medial prefrontal cortex
dHC: Dorsal hippocampus
S1: Primary sensory cortex
DG: Dentate gyrus
CA3: Cornu ammonis 3
CA1: Cornu ammonis 1
dCLNs: Deep cervical lymph nodes
AQP4: Aquaporin-4
GFAP: Glial fibrillary acidic protein
IBA1: Ionized calcium-binding adapter molecule 1
i.c.m.: Injection into the cisterna magna
WT-Ctrl: Wild type littermates received sham treatment
AD-Ctrl: 5xFAD mice received sham treatment
AD-rTMS: 5xFAD mice received rTMS treatment
SSS: Superior sagittal sinus
TS: Transverse sinus
SEM: Standard error of the mean
