# Supplementary figures and images for: Repetitive transcranial magnetic stimulation increases the brain’s drainage efficiency in a mouse model of Alzheimer’s disease
Source: Acta Neuropathol Commun. 2021 Jun 2;9:102. doi: 10.1186/s40478-021-01198-3 (PMC8170932; doi:10.1186/s40478-021-01198-3)

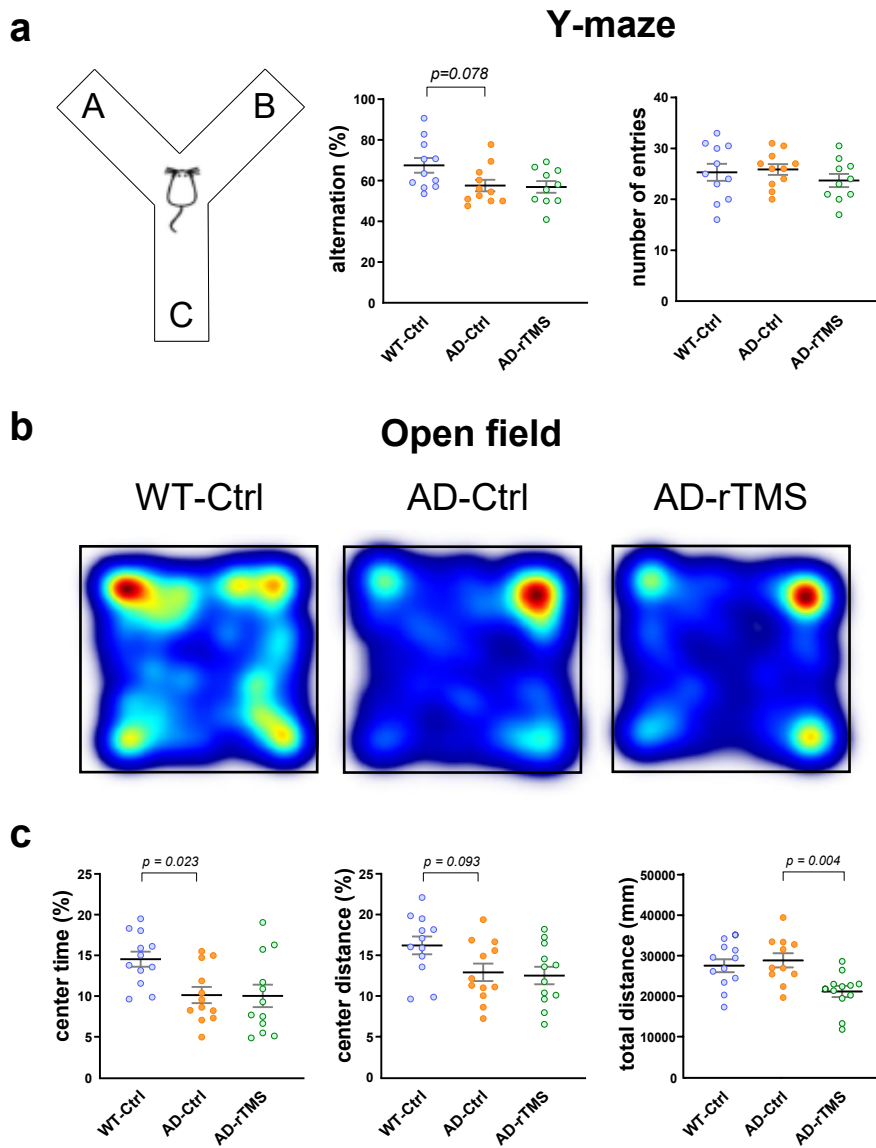

**Supplementary Figure 1**

Supplement: Supplementary file 1 — Additional file 1: Supplementary Fig. 1. The effect of rTMS on spatial working memory and anxiety-like behaviors. a. Left: schematic of the Y-maze task. A, B and C are the 3 arms of the Y-maze. Right: Quantitative analyses of the spontaneous alternation (% alternation) and the number of total arm entries across groups (n = 10-11 mice per group). b. Representative heatmaps of animals’ paths in the open field test. c. Quantitative analyses of the time spent in the center zone as percentage of the total time, the distance travelled in the center zone as percentage of the distance travelled, and the total distance travelled in the open field across groups (n = 11-12 per group). All data are presented as mean ± SEM and analyzed by one-way ANOVA followed by Tukey's multiple comparisons test. WT-Ctrl: wildtype littermates received sham treatment, AD-Ctrl: 5xFAD mice received sham treatment, AD-rTMS: 5xFAD mice received rTMS treatment. [file 40478_2021_1198_MOESM1_ESM.pdf]

**a**

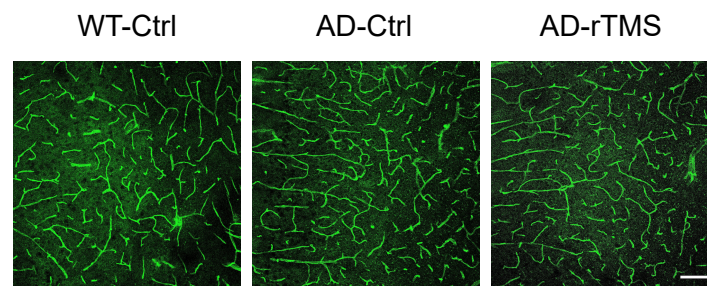

**b**

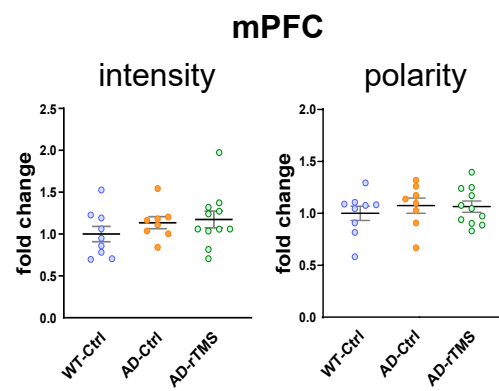

**Supplementary Figure 2**

Supplement: Supplementary file 2 — Additional file 2: Supplementary Fig. 2. rTMS does not change AQP4 expression or polarization in the mPFC. a. Representative images of AQP4 staining in the medial prefrontal cortex (mPFC). Scale bar: 150 μm. b. Quantitative analyses of the fluorescence intensity (normalized to the area of mPFC) of AQP4 staining and AQP4 polarity (defined as the fluorescence intensity of AQP4 staining on the perivascular end divided by the fluorescence intensity of total AQP4 staining) in the mPFC across groups (n = 8-9 mice per group). All data are presented as mean ± SEM of the fold change of the WT-Ctrl group and analyzed by one-way ANOVA followed by Tukey's multiple comparisons test. WT-Ctrl: wildtype littermates received sham treatment, AD-Ctrl: 5xFAD mice received sham treatment, AD-rTMS: 5xFAD mice received rTMS treatment. [file 40478_2021_1198_MOESM2_ESM.pdf]
